# Importance of additional behavioral observation in psychopharmacology: a case study on agomelatine’s effects on feedback sensitivity in probabilistic reversal learning in rats

**DOI:** 10.1007/s00213-023-06443-2

**Published:** 2023-08-12

**Authors:** Karolina Noworyta, Agata Cieslik-Starkiewicz, Rafal Rygula

**Affiliations:** Affective Cognitive Neuroscience Laboratory, Department of Pharmacology, Maj Institute of Pharmacology Polish Academy of Sciences, Smetna 12, 31-343, Krakow, Poland

**Keywords:** Negative feedback sensitivity, Animal model, Behavior, Rat, Agomelatine

## Abstract

Since the second half of the twentieth century, many important discoveries in the field of behavioral psychopharmacology have been made using operant conditioning cages. These cages provide objective data collection and have revolutionized behavioral research. Unfortunately, in the rush towards automation, many mistakes may have been made that could have been avoided by observing experimental animals. The study described in this paper is an excellent example of how important additional behavioral observation can be for interpreting instrumental data. In this study, we evaluated the effects of single injections of 3 different doses of agomelatine (5, 10, and 40 mg/kg) on feedback sensitivity in rats. To this end, we tested 40 animals in the instrumental probabilistic reversal learning task in a Latin square design. The highest applied dose of agomelatine, prima facie, reduced the sensitivity of rats to negative feedback — an effect that can be considered antidepressant. However, additional behavioral observation dramatically changed the interpretation of the results and revealed that the perceived effect of agomelatine on sensitivity to negative feedback can actually be attributed to drug-induced drowsiness.

## Introduction

From the very beginning of our scientific careers, we are trained to record, sort, interpret, and analyze numbers. This makes us steadfast in our pursuit of expressing every phenomenon in the form of numbers, and the numerical notation is widely recognized as a universal way of documenting and publishing the results of experimental observations (Chen and Sarma 2019; Newman 2019). Thanks to constantly evolving mathematical skills, the most adept scientists are even able to describe the behavior of laboratory animals in the form of a neat equation (Daw 2011).

Although in the behavioral sciences the numbers provide a sense of objectivity and reliability, there have been numerous cases throughout the history of scientific discovery where the numerical recording of the behavioral data has proved insufficient. One such case, which is particularly close to the authors of this paper, is the story of Professor Jerzy Vetulani (Vetulani and Sulser 1975), during the early days of the scientific community’s fascination with the miraculous properties of lysergic acid diethylamide (LSD) and its derivatives. In the 1970s, a young Vetulani conducted research on the effects of LSD on the locomotion of rats. The results from the automatized apparatus used to measure the animals’ mobility, unequivocally indicated that both control and LSD-injected rats ran at the same speed and covered a similar distance. However, the scientist’s curiosity outweighed his disappointment with the obtained numerical data, and he decided to observe how the animals were behaving. It turned out that rats given LSD were running backwards. Although these observations were not publishable, they have survived to this day as an anecdote, and they carry a valuable lesson: in behavioral neuroscience research, quantitative and qualitative data are equally important, and neither should be overlooked (Slagter and Bouwer 2021).

Indeed, qualitative observations are particularly important during the execution of behavioral tests measuring higher cognitive functions, such as sensitivity to feedback or cognitive flexibility. One such test is the probabilistic reversal learning (PRL) test, which is used to assess sensitivity to performance feedback in both human patients (Roiser et al. 2009) and experimental animals (Rygula et al. 2018).

Individuals with depression often engage in rumination, excessively focusing on perceived failures and criticism (Spasojevic and Alloy 2001). Growing research evidence from studies employing PRL paradigms indicates that individuals with depression exhibit an amplified response to negative feedback (NF) and reduced sensitivity to positive feedback (PF) during laboratory testing (Mukherjee et al. 2020; Robinson et al. 2012). While studies conducted in the past two decades have provided some understanding of the neural basis underlying the altered sensitivity to feedback in depression, the neurochemical correlates of this process are still not fully comprehended. The successful application of the PRL paradigm in rats, as demonstrated by Bari et al. (2010), has opened new avenues for studying the neural substrates and molecular mechanisms associated with feedback processing in preclinical animal models.

In the preclinical version of this task, the rats are tested in cages for instrumental conditioning, allowing automatic recording of several quantitative variables, including the numbers of win-stay (WS) and lose-shift (LS) behaviors following the true and misleading PF and NF. Attention of the experimenter during the test sessions should also allow for the recording of qualitative variables, such as how the animals behave when placed in the cage, their state of arousal, whether they interact, and how, with the elements of the cage (feeders, levers).

Although several previous studies demonstrated that various serotonergic manipulations (e.g., acute and chronic treatment with the selective serotonin reuptake inhibitor — citalopram) are effective in the modulation of sensitivity to feedback in rats performing the PRL (Bari et al. 2010), and that acute injection of the NMDA receptor antagonist — ketamine, can reduce sensitivity to NF (Rychlik et al. 2017), the effects of other antidepressants within this domain remain still uninvestigated.

The aim of this study was to test the hypothesis that a single dose of agomelatine (Ago), one of the most commonly used new-generation antidepressants, could modulate the sensitivity to NF and PF in rats. Due to its unique pharmacological profile, including its affinity for serotonin 5-HT_2c_ and melatonin M_1_ and M_2_ receptors, Ago has previously been postulated to be highly effective in the antidepressant therapy (Descamps et al. 2009; Guardiola-Lemaitre et al. 2014; Millan et al. 2003), and its effects on sensitivity to feedback could provide insights into the mechanism of its antidepressant action.

The hypothesized effects of Ago on feedback sensitivity are grounded inter alia, in the findings of studies conducted by Phillips et al. (2018). These studies demonstrated that the responsiveness to PF can be influenced through pharmacological modulation of the serotonin receptor subtype 5-HT_2C_. Specifically, in this study, the administration of SB 242084, an antagonist of the 5-HT_2C_ receptor, led to diminished sensitivity to PF. Conversely, the administration of WAY 163909, an agonist of the abovementioned receptor, produced alterations linked to heightened sensitivity to PF and reduced sensitivity to NF.

Interestingly, although the analysis of the quantitative data obtained from our experiments suggested that Ago reduces the sensitivity of animals to NF — an effect that could be interpreted as antidepressive, the additional, careful observation of animals’ behavior during the experiment revealed a completely different result. It turned out that the apparently reduced sensitivity to NF was due to the increased drowsiness of the animals after administration of Ago rather than the effects of the drug on sensitivity to feedback.

## Methods

### Ethics statement

All experiments were conducted following the European Union guidelines for the care and use of laboratory animals (2010/63/EU). Experimental protocols were reviewed and approved by the 2nd Local Institutional Animal Care and Use Committee at the Maj Institute of Pharmacology Polish Academy of Sciences in Krakow (Permission no. 242/2017).

### Subjects

We used 40 male Sprague–Dawley rats (Charles River, Germany) weighing 175–200 g upon arrival. Rats were kept in groups (4 animals per cage, randomly assigned) under controlled temperature (21 ± 1 °C) and humidity (40–50%) conditions. Animals were housed at a 12/12 h light/dark cycle (lights on at 7:00 h). During the whole experiment, rats were mildly food restricted to 85% of their free-feeding weight (according to the normal growth curve recommended by the laboratory rodent supplier — Charles River Research Models and Services Catalogue) by providing 15–20 g of food pellet per rat per day (standard laboratory chow). Food restriction began 1 week prior to behavioral training. Water was available ad libitum. All of the conducted behavioral procedures and tests were performed during the light phase of the light/dark cycle.

### Drug administration

Ago (TCI, Zwijnderecht, Belgium) was suspended in 1% hydroxyethyl cellulose and administered intraperitoneally (i.p.) in a dose volume of 1 ml/kg, 30 min before the PRL test sessions using fully randomized Latin square design. The drug was administered in 3 different doses: 5, 10, and 40 mg/kg. The wash-out period between administrations of different doses in the Latin square design was 1 week. An example of Latin square design is presented in Table 1. Dose range was chosen based upon previous documented effects in various animal models (Drozd et al. 2019; Lan et al. 2022; Papp et al. 1993).

**Table 1:** Latin square design used for agomelatine administration

Dose/test	1	2	3	4
1	Vehicle	10 mg/kg	5 mg/kg	40 mg/kg
2	5 mg/kg	Vehicle	40 mg/kg	10 mg/kg
3	10 mg/kg	40 mg/kg	Vehicle	5 mg/kg
4	40 mg/kg	5 mg/kg	10 mg/kg	Vehicle

### Measuring reinforcement sensitivity using the PRL test

The PRL tests were performed in 8 computer-controlled operant conditioning boxes (Med Associates; St Albans, Vermont, USA), each equipped with a light, fan, speaker, a food dispenser set to deliver a sucrose pellet (Dustless Precision Pellets, 45 mg; Bio-Serv, NJ, USA), and 2 retractable levers located at the opposite sides of the feeder. All applied behavioral protocols were programmed in Med State notation code (Med Associates) and analyzed with a custom-written R program. The training procedure for the PRL task used in this study was a modified version of the procedures used and described previously by Bari et al. (2010) and has been described in detail elsewhere (Noworyta-Sokolowska et al. 2019; Rychlik et al. 2017). A figure demonstrating task procedure has been already published elsewhere (Rychlik et al. 2017).

In brief, each PRL training session consisted of 200 trials, and each trial lasted for a maximum of 22 s. The start of a trial was signaled by the house light, which remained on until the end of the trial. Two seconds after the trial started, both levers were extended and the one that was pressed first became the “correct” lever which delivered the reward (sucrose pellet) with 80% probability. During the reward delivery, the levers retracted, and the house light remained on for 5 s. The same intertrial interval (ITI) directly followed an unrewarded outcome, i.e., no reward on 20% of the “correct” lever presses. In the following trial, pressing the “correct” lever would again result in reward delivery with 80% probability, while pressing the other lever — the “incorrect” lever — would result in a rewarding outcome with a probability of only 20%. No response in 10 s triggered the fixed 5 s ITI and was counted as an omission. During the ITI, both levers remained retracted, and the house light was switched off. After every 8 consecutive “correct” lever presses (regardless of the outcome), the criterion for the reversal of the outcome probabilities was reached. The previously “correct” lever now became “incorrect” and vice versa. This pattern was followed until the end of the session.

This training phase was repeated daily until the individual animals achieved sufficient performance levels. The criteria to be met were a minimum of 3 reversals completed during 3 consecutive training sessions, with less than 15% omissions per session. All animals fulfilled the training criteria and qualified for further testing and drug administration. On average, the animals reached the criteria after 8 ± 4 PRL tests.

### Parameters measured in the PRL test

For measurement of feedback sensitivity in the PRL test, we have used procedures previously described elsewhere (Drozd et al. 2019; Noworyta-Sokolowska et al. 2019; Rychlik et al. 2017). In brief, the animals’ decisions were tracked trial-by-trial, which allowed for data collection on a similar basis. To evaluate the ability of animals to ignore infrequent and misleading NF, non-rewarded outcomes on the “correct” lever after which an animal decided to switch levers (probabilistic LS) were scored and expressed as a ratio of all non-rewarded outcomes on that lever. To assess animals’ ability to respond to NF, non-rewarded outcomes on the “incorrect” lever after which an animal decided to switch lever (actual LS) were scored and expressed as a ratio of all non-rewarded outcomes on that lever. The sensitivity to PF was calculated analogously. To assess sensitivity to PF, all rewarded outcomes followed by a decision to stay with the lever that delivered them (WS) were counted jointly for the “correct” and “incorrect” levers and expressed as a ratio of all rewarded outcomes on that lever.

The number of reversals completed during the test was used to assess cognitive flexibility, which relies on suppressing previously rewarded actions and engaging in previously unrewarded actions (Nilsson et al. 2015). The latency to press the lever and the number of omissions was measured for all trials regardless of the outcome (not distinguishing between those following the receipt of a reward or a punishment) as a measure of the general performance of the animals.

### Measuring sensitivity to feedback and cognitive flexibility following Ago administration

Thirty minutes after Ago administration, a single PRL test was performed. The proportion of probabilistic LS and WS behaviors as well as the number of reversals made, the latency to press the lever, and the number of omissions was measured.

### Statistical analysis

The data were analyzed using GraphPad Prism (version 9.5.0, GraphPad Software, Inc., San Diego, CA). The normality of the data distribution was confirmed using the Kolmogorov–Smirnov test. The effects of Ago on sensitivity to NF and PF were investigated using repeated-measures analysis of variance (ANOVA) with the within-subjects factor of dose (four levels: vehicle, dose 1, dose 2, and dose 3). For pairwise comparisons, the values were adjusted using Sidak’s correction factor for multiple comparisons (Howell 1997). The data that did not meet the criteria of normal distribution (number of reversals, latency to press the lever, number of omissions) were analyzed using the Friedman test followed by a Dunn’s post hoc test. All significance tests were performed at α = 0.05. For repeated-measures analyses, sphericity was verified using Mauchly’s test.

## Results

### Lose-shift performance

ANOVA revealed statistically significant effect of Ago on LS behavior following misleading (*F*_*(*3,117)_ = 14.88, *p* < 0.0001) and actual (*F*_(3,117)_ = 18.22, *p* < 0.0001) NF.

**Fig. 1:**
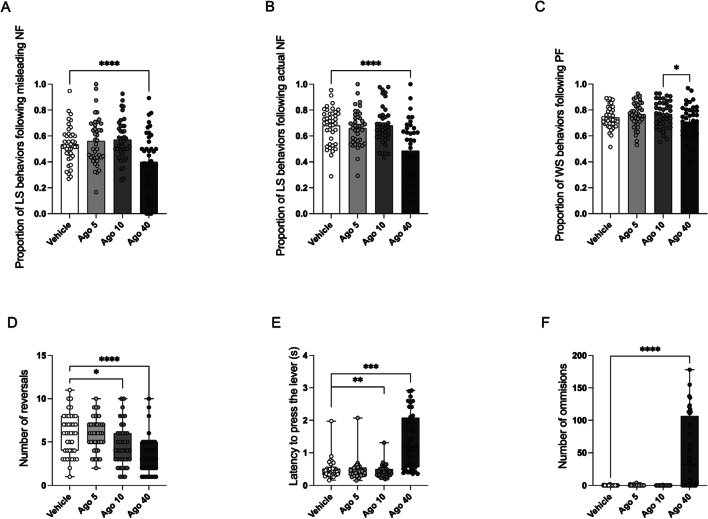
Effects of acute administration of 3 different doses (5, 10, and 40 mg/kg) of agomelatine (Ago) on rats’ performance in the PRL test. A and B demonstrate the effects of Ago on the proportion of lose-shift (LS) behaviors following misleading and actual negative feedback (NF), respectively. C shows the effects of Ago on the proportion of win-stay (WS) behaviors following positive feedback (PF). D, E, and F display the effects of Ago on reversal performance, latency to press the lever, and omissions made by animals during PRL tests, respectively. The data are presented as mean ± SEM (A, B, and C) or as a median and interquartile range (D, E, and F); *p < 0.05, **p < 0.01, ***p < 0.001, ****p < 0.0001, *N* = 40 for each group

Ago at the highest tested dose (40 mg/kg) significantly decreased the proportion of LS behaviors following misleading (*t* = 4.62, *p* < 0.0001) and actual (*t* = 6.20, *p* < 0.0001) NF, as compared to the vehicle-treated controls (Fig. 1A and B, respectively). At the doses of 5 and 10 mg/kg, Ago administration had no statistically significant effects on the proportion of LS behaviors following neither the misleading (*t* = 0.90, *p* = 0.9386, and *t* = 1.28, *p* = 0.7411, respectively) or actual (*t* = 0.58, *p* = 0.9932, and *t* = 0.02, *p* > 0.9999, respectively) NF (Fig. 1A and B, respectively) in comparison to the vehicle-treated controls.

### Win-stay performance

Although the ANOVA revealed statistically significant effect of dose (*F*_(3,117))_ = 3.70, *p* = 0.0137) on the proportion of WS behaviors, post hoc comparisons confirmed statistically significant difference only between the groups of animals receiving the Ago doses of 10 and 40 mg/kg (*t* = 3.09, *p* = 0.0151, Fig. 1C).

None of the tested doses of Ago had statistically significant effects on the proportion of WS behaviors following PF as compared to vehicle (*t* = 0.94, *p* = 0.9257; *t* = 1.42, *p* = 0.6479; and *t* = 1.67, *p* = 0.4588, for the doses of 5, 10, and 40 mg/kg, respectively, Fig. 1C).

### Reversal performance

Ago administration significantly affected the reversal performance of experimental animals (chi-square = 39.73, *p* < 0.0001, Fig. 1D). The rats that received the doses of 10 and 40 mg/kg made significantly (*Z* = 2.86, *p* = 0.0256, and *Z* = 4.98, *p* < 0.0001, respectively) fewer reversals than the vehicle-treated controls. This effect was not observed for the lowest tested dose (*Z* = 0.04, *p* > 0.9999, Fig. 1D).

### Latency to press a lever

Ago also had statistically significant effect on the latency to press the lever (chi-square = 70.05, *p* < 0.0001). The rats that received Ago at the dose of 10 mg/kg significantly (*Z* = 3.64, *p* = 0.0017) reduced, while the ones treated with Ago at the dose of 40 mg/kg significantly (*Z* = 4.07, *p* < 0.0003) increased the latency to press a lever in comparison to the vehicle-treated controls (Fig. 1E). Ago, at the lowest tested dose (5 mg/kg), had no significant (*Z* = 2.51, *p* = 0.0721) effects on the latency to press a lever (Fig. 1E).

### Omissions

Last but not least, Ago had statistically significant effect on the number of response omissions (chi-square = 73.06, *p* < 0.0001, Fig. 1F). The rats treated with Ago at the dose of 40 mg/kg made significantly (*Z* = 4.89, *p* < 0.0001) more omissions than the vehicle-treated controls. At the lower tested doses of 5 and 10 mg/kg, Ago administration had no statistically significant (*Z* = 0.39, *p* > 0.9999, and *Z* = 0.09, *p* > 0.9999, respectively) effects on this parameter (Fig. 1F).

## Discussion

The results of the conducted experiments suggest that at the highest tested dose, Ago reduces the sensitivity of animals to NF. This can be inferred from the reduced proportion of LS behaviors following administration of the drug. The effects of Ago appear to be specific to sensitivity to NF, as no statistically significant change was observed in the proportion of WS behaviors. Moreover, the administration of Ago was also accompanied by a reduced number of reversals, an increased latency to press a lever, and a significantly increased number of omissions.

At first glance, these results seem to fit together nicely, suggesting that Ago acts selectively on the sensitivity of animals to NF. Although the animals were slower and more likely to omit trial when administered the highest dose of Ago, the lack of effect on PF seems to control for the drug’s effect on sensitivity to NF. These results seem to be also consistent with the hypothesis that antidepressant drug administration modulates cognitive processing very early in treatment (Harmer et al. 2009), before changes occur in mood or other symptoms and are consistent with cognitive models of depression that emphasize the important role of correction of cognitive distortions in effective pharmacotherapy (Harmer et al. 2003).

However, this interpretation is not entirely accurate. Additional observations of the animals lead to completely different conclusions. Under the influence of a high dose of Ago, the animals appeared drowsy and tended to stay near the nearest lever in the operant conditioning cages. Once pressed, this lever became the “correct” lever, and pressing it was associated with an 80% chance of receiving a reward. Despite their drowsiness, the animals persisted in pressing this lever, albeit slowly and with many omissions. This resulted in a lack of LS behaviors typical during the exploratory phase of this discrimination. When the criterion was eventually reached and the animals received NF, they persevered by continuing to press the same lever instead of switching to the other lever. This was reflected in the reduced proportion of LS behaviors. As the animals overcame their drowsiness and switched levers, the entire process repeated itself, resulting in another reduction in the LS ratio. The described mechanism also explains why the proportion of WS behaviors remained unchanged, even with a high frequency of omissions. This proportion is calculated in relation to all lever presses that occur after PF but excludes omissions. In animals receiving the high dose of Ago, it was simply calculated from a smaller number of lever presses.

Although the dose of Ago 40 mg/kg appears to be clearly too high, as evidenced by the observed drowsiness, it was chosen based on several previous publications reporting its effectiveness in reducing symptoms of depression in animal models (Haduch et al. 2020; Rossetti et al. 2022). Interestingly, none of these publications mentioned the increased sleepiness of the animals observed in our experiment, perhaps due to the lack of additional qualitative, behavioral observation. The observed drowsiness may result from the specific mechanism of Ago action, which is based on the antagonism of serotonin receptors 5-HT_2C_, as well as the agonism of melatonin M_1_ and M_2_ receptors (Millan et al. 2003; Racagni et al. 2011). Because Ago has a stronger affinity for melatonin receptors than for serotonin receptors (Guardiola-Lemaitre et al. 2014), the activation of the former can affect the sleep–wake cycle in animals. Indeed, stimulation of melatonin receptors by Ago has been linked to an improvement (advanced onset) of sleep and circadian rhythms both in humans and animals (Descamps et al. 2009; Outhoff 2012).

The increased latency to press a lever following the administration of Ago at a dose of 40 mg/kg has previously been reported by Drozd et al. (2019). In our previous studies, we have also demonstrated that the effects of a single administration of Ago do not interact with the trait sensitivity to feedback and that single administration of this drug does not impact the hedonic status of rats as measured in the sucrose preference test (Surowka et al. 2020). To the best of our knowledge, no other studies have investigated the effects of Ago on the PRL.

### Limitations

While our study contributes to our understanding of the effects of Ago on sensitivity to feedback, the exclusive use of male subjects represents a potential limitation. The lack of females limits the generalizability of our findings to a broader population, particularly in the context of gender-specific effects and depression. Future research should aim to address this limitation by including both males and females, enabling a more comprehensive understanding of the nuanced effects of Ago on sensitivity to feedback. Another limitation of our study is the absence of systematically quantified behavioral observations. Undoubtedly, the systematic quantification of qualitative parameters such as drowsiness would significantly strengthen our conclusions. Regrettably, such observations were not planned before the start of the experiment and were made ad hoc during the course of our study, resulting in a lack of quantifiable data. Nonetheless, we maintain a strong belief that the case described in our manuscript can contribute to minimizing interpretation errors and serve as inspiration for future researchers to incorporate an additional, quantifiable system for evaluating the behavior of animals involved in operant conditioning paradigms.

## Conclusions

Incorporating qualitative measures into behavioral neuroscience studies can be beneficial for a comprehensive understanding of animal behavior. One possible approach is to move towards the adoption of more standardized scales for qualitative assessments. By establishing common metrics and criteria, researchers can ensure consistency and comparability across studies. This not only facilitates data analysis and interpretation but also helps prevent the oversight of critical observations. Emphasizing the importance of standardized qualitative measures in the field can encourage their widespread implementation, enabling researchers to capture and document crucial behavioral nuances consistently across diverse studies.

The results and observations described in this study may contribute to a change in approach to obtaining data using advanced behavioral paradigms based on numerical data generated automatically or semi-automatically. Undoubtedly, the advantage of such paradigms is the minimization of the risk of experimenter bias towards favoring results that allow for the rejection of the null hypothesis of no differences between groups. However, the example of this study shows that they are not without serious flaws. Automating the acquisition of quantitative data is entirely justified in studies using cells, tissues, or organs. However, in studies using living organisms, the complete omission of qualitative or descriptive data can lead to false conclusions. That is why it is so important when planning behavioral experiments not to limit oneself only to putting the animal into a “black box that spits out numerical data.” Following observations by an experienced experimenter behaviorist, the interpretation of the obtained results could be radically different.

## Data Availability

All data analyzed in this study have been made publicly available and can be accessed through DOI: 10.5281/zenodo.8071104
